# Exploring New Functional Aspects of HTLV-1 RNA-Binding Protein Rex: How Does Rex Control Viral Replication?

**DOI:** 10.3390/v14020407

**Published:** 2022-02-16

**Authors:** Kazumi Nakano, Koichi Yokoyama, Shuichi Shin, Koki Uchida, Kazuki Tsuji, Marie Tanaka, Kaoru Uchimaru, Toshiki Watanabe

**Affiliations:** 1 Department of Computational Biology and Medical Sciences, Graduate School of Frontier Sciences, The University of Tokyo, Tokyo 108-8639, Japan; 2 Department of Practical Management of Medical Information, Graduate School of Medicine, St. Marianna University, Kanagawa 216-8511, Japan

**Keywords:** HTLV-1 Rex, interactome, transcriptome, alternative splicing analysis, viral genome expression, PD-L1, NONO/SFPQ

## Abstract

After integration to the human genome as a provirus, human T-cell leukemia virus type 1 (HTLV-1) utilizes host T cell gene expression machinery for viral replication. The viral RNA-binding protein, Rex, is known to transport unspliced/incompletely spliced viral mRNAs encoding viral structural proteins out of the nucleus to enhance virus particle formation. However, the detailed mechanism of how Rex avoids extra splicing of unspliced/incompletely spliced viral mRNAs and stabilizes them for effective translation is still unclear. To elucidate the underlying molecular mechanism of Rex function, we comprehensively analyzed the changes in gene expression and splicing patterns in Rex-overexpressing T cells. In addition, we identified 81 human proteins interacting with Rex, involved in transcription, splicing, translation, and mRNA quality control. In particular, Rex interacts with NONO and SFPQ, which play important roles in the regulation of transcription and splicing. Accordingly, expression profiles and splicing patterns of a wide variety of genes are significantly changed in Rex-expressing T cells. Especially, the level of v*PD-L1* mRNA that lacks the part of exon 4, thus encodes soluble PD-L1 was significantly increased in Rex-expressing cells. Overall, by integrated analysis of these three datasets, we showed for the first time that Rex intervenes the host gene expression machinery throughout the pathway, probably to escort viral unstable mRNAs from transcription (start) to translation (end). Upon exerting its function, Rex may alter the expression level and splicing patterns of various genes, thus influencing the phenotype of the host cell.

## 1. Introduction

HTLV-1 (human T cell leukemia virus type 1) is transmitted to human T cells by mother-to-child transmission, mainly through breast milk, or by horizontal transmission through blood transfusion or sexual intercourse. After a long incubation period of 50–60 years, about 5% of infected individuals develop ATLL (adult T-cell leukemia/ lymphoma), and 0.3% develop HAM (HTLV-1-associated myelopathy) or HU (HTLV-1-associated uveitis). HTLV-1 is a single-stranded, positive-sense RNA virus belonging to the genus *Deltaretrovirus* in the family *Retroviridae*. After reverse transcription, HTLV-1 is permanently integrated into the human genomic DNA of host T cells as a 9 kb provirus with long terminal repeats (LTRs) at both ends, and viral replication occurs through the host cell′s transcription and translation machinery. Fully spliced *Tax/Rex* mRNA is first transcribed from the 5′-LTR and *HBZ* (HTLV-1 basic region leucine-zipper) mRNA from the 3′-LTR. Tax specifically and potently activates the 5′-LTR and promotes transcription of unspliced Gag/Pro/Pol-encoding and once (incompletely) spliced Env-encoding mRNAs, which are nuclear-exported by Rex. HBZ acts antagonistically with Tax to negatively regulate the transcription of viral genes, while increasing the efficiency of HTLV-1 infection in vivo and promoting the growth of infected T cells [1,2,3]. The coordinated actions of these viral accessory proteins are thought to regulate viral replication in the early stages of HTLV-1 infection and the subsequent transition to a stable latent infection period, but the reason why HTLV-1 causes different diseases is not known. The relationship between events in infected cells and the fate of infected cells during the subsequent long latent infection period has not been clarified.

Rex, the mRNA-binding protein of interest in this study, binds specifically to the higher-order RxRE (Rex-responsive element), which is present in the 3′-U3/R region of all HTLV-1 mRNA variants. Rex also binds to the cellular nuclear export protein CRM1 to stably transport those unstable viral mRNAs out of the nucleus (see review by Nakano and Watanabe [4,5]). The specialized Rex/CRM1 complex-dependent mRNA nuclear export enables the translation of unspliced and incompletely spliced viral mRNAs, encoding viral structural proteins that are not normally present in the cytoplasm. In turn, the translation of *Tax/Rex* mRNA, which is dependent on the regular CAP-dependent nuclear export, is reduced. Consequently, Tax and Rex function and virus particle formation are suppressed, leading to a chronic latent state in infected cells. Thus, the transition from active viral replication to latency in the early phase of HTLV-1 infection is precisely regulated by the balance of Tax and Rex activities. In particular, changes in the extracellular trafficking of unspliced and incompletely spliced viral mRNA by Rex regulate the efficiency of viral particle replication and the timing of the transition to latency [6]. However, the detailed molecular mechanisms by which Rex performs its function, such as preventing further splicing in unspliced Gag/Pro/Pol-encoding and once-spliced Env-encoding mRNAs, remain largely unexplored. We found that HTLV-1 unspliced (genomic) mRNA is a target of host nonsense-mediated mRNA decay (NMD), and it was reported that Rex suppresses NMD and stabilizes viral mRNA [7]. There are reports suggesting that Rex may act on host splicing machinery [8,9], but the mechanism remains to be elucidated. We hypothesize that Rex may have as-yet-undiscovered functions and effects in infected cells to regulate the efficiency of viral particle replication through selective extranuclear trafficking of HTLV-1 unspliced/incompletely spliced mRNAs. However, knowledge of the molecular characteristics of Rex, such as whether Rex changes the expression level and the splicing pattern of host T-cell mRNAs, is far from enough to understand the functional mechanism of Rex. It is well known that Rex interacts with cellular CRM1 and importin-b via its nuclear export signal (NES) and nuclear localization signal (NLS), respectively. However, comprehensive examination of human proteins interacting with Rex has not been fully accomplished. There is a significant lack of information about Rex in order to understand the full range of its functions and molecular mechanism at the site of HTLV-1 infection in T cells. In this study, we performed a comprehensive analysis in transcriptome, alternative splicing, and interactome of Rex, and integrated these datasets to clarify how Rex acts and functions in human T cells. 

## 2. Materials and Methods

### 2.1. Transcriptome and Alternative Splicing Analysis in Rex-Expressing CEM Cells

#### 2.1.1. Construction of Retroviral Plasmid for Rex Expression

Rex-pME-FLAG [7] was used as a template, and the flowing primers were used to amplify the Rex cDNA fragment by PCR (Platinum Taq High Fidelity, Invitrogen, Thermo Fisher Scientific, Inc., Waltham, MA, USA).

P27Rex(EcoRI)-For: 5′-GAAATTCTCGCCACCATGCCCAAGACCCG-3′

P27Rex(NotI)-Rev: 5′-GCGGCCGCTCACATGGGGCAGGAG-3′

The Rex fragment was inserted into the EcoRI/NotI site of a retrovirus vector pRx-Puro [10] to create pRx-Puro-Rex.

#### 2.1.2. Establishment of Rex-Expressing CEM Using Retrovirus Vector Expression System

After 24 h, HEK293FT cells were seeded at 5 × 10^5^ cells/6 cm dish with 5 μg of retrovirus gag/pol vector, 5 μg of env vector, and 10 μg of pRx-Puro (Mock) or pRx-Puro-Rex by calcium phosphate method. The culture medium was changed after 4 h, and after 48 h the supernatant was filtered through a 0.45 μm filter and used as viral solution. CEM cells (human TALL patient-derived T cell line) were cultured in the viral solution to be infected with the recombinant retrovirus expressing Rex or no protein (Mock). After 48 h, puromycin selection (0.5 μg/mL) was started, and the established cells (CEM-Rex and CEM-Control) were used for experiments after 10–12 days.

#### 2.1.3. Gene Expression and Exon Microarray Analysis

RNA was extracted using TRI-Zol (Invitrogen, Thermo Fisher Scientific, Inc., Waltham, MA, USA) from CEM-Rex and CEM-Control (*n* = 4 each) and subjected to the following microarray analysis. For gene expression microarray, the Cy3 labeling of total RNA was performed using Low Input Quick Amp Labeling Kit, and the prepared cRNA (complementary RNA) sample was hybridized with the gene expression microarray (Human GE 4 × 44K V2 Microarray Kit, #G4845A, Agilent Technologies, Inc., Santa Clara, CA, USA). After 17 h of hybridization at 65 °C, the microarrays were scanned by Scanner C (Agilent Technologies Inc., Santa Clara, CA, USA) and the signals were quantified by Feature Extraction v10.7.3 (Agilent Technologies Inc., Santa Clara, CA, USA). Data normalization and statistical analysis of those datasets were performed using Gene Spring 14.4 (Agilent Technologies Inc., Santa Clara, CA, USA).

For exon microarray analysis, the Cy3 labeling was also performed using the Low Input Quick Amp WT Labeling Kit and hybridized to the exon microarray (SurePrint G3 Human Exon 2 × 400 K Microarray Kit, #G4848A, Agilent Technologies Inc., Santa Clara, CA, USA). After 17 h of hybridization, the samples were scanned by Scanner C (Agilent Technologies Inc., Santa Clara, CA, USA) and the signals were quantified by Feature Extraction v10.7.3 (Agilent Technologies Inc., Santa Clara, CA, USA). The human exon microarray used in this study is equipped with probes (approximately 400,000 probes) for each exon of total human mRNA. Splicing index (SI), which is calculated as the ratio between the expression of an exon (exon probe intensity) and the expression level of the mRNA in which the exon is involved, indicates loss or amplification of the exon within the mRNA. For example, the SI of exon 1 for a given mRNA-A is expressed as the ratio between the level of exon 1 relative to total mRNA-A expression in CEM-Rex and CEM-Control. If SI is significantly different in CEM-Rex compared with CEM-Control, then the exon is differentially expressed, and the mRNA is alternatively spliced in CEM-Rex. Based on the normalized exon probe intensity values derived from Gene Spring 14.4 (Agilent Technologies Inc., Santa Clara, CA, USA), we manually calculated SI in each exon in CEM-Rex and CEM-Control using Microsoft Excel software (Microsoft Corp., Redmond, WA, USA). Then, we performed *F*-test followed by Student’s *t*-test on probe intensity values of an exon between CEM-Rex and CEM-Control (*n* = 4 each). If the SI of one or more exons in an mRNA is significantly different (*p* < 0.05) between CEM-Rex and CEM-Control, the mRNA is defined as alternatively spliced mRNA in CEM-Rex.

#### 2.1.4. Biological Annotation Analysis of the Microarray Data and the Interactome Data

In the present study, the biological significance of the data that were obtained from microarray analysis (described in Section 2.1) and interactome data (described in Section 2.2) were analyzed using STRING (v11.5) [11]. Briefly, the whole dataset of gene expression microarray analysis, i.e., data of all probes with expression levels (CEM-Rex against CEM-Control), were input to “Protein with Value/Ranks” application of STRING (v11.5) to perform the Functional Enrichment Analysis (FEA, hereafter). The results were returned as lists of terms in various databases such as Gene Ontology, KEGG, Pathways, Reactome, COMPARTMENTS, UniProt, Pfam, and InterPro, which are significantly enriched with our gene expression data. Since our data include information on gene expression levels, terms with most genes that are higher in CEM-Rex than in CEM-Control are considered as “upregulated”, while terms with most genes that are lower in CEM-Rex than in CEM-Control are considered as “downregulated”. For the exon microarray data, the list of mRNAs that were alternatively spliced in CEM-Rex compared with CEM-Control, i.e., the probe intensity was significantly different (*p* < 0.05) between CEM-Rex and CEM-Control in more than one probe (exon) within an mRNA, was also analyzed in “Protein with Value/Ranks” application of STRING (v11.5) without the information of values (expression levels). Finally, regarding the Rex interactome data, the list of proteins that interact with Rex was analyzed in “Protein by Name” application of STRING (v11.5) to perform FEA for a small number of inputs. 

### 2.2. Rex Interactome Analysis

#### 2.2.1. Preparation of His-Halo-Rex Expression Plasmid

From Rex-pME-FLAG [7], the Rex fragment was cut out at EcoRI and XbaI sites and subcloned into PHTN-Halo Tag CMV-neo vector (Promega, Corp., Madison, WI, USA). In addition, a 6× His tag sequence was inserted upstream of the Halo tag sequence.

#### 2.2.2. Identification of Interacting Proteins by Tandem Affinity Purification of His-Halo-Rex

HEK293FT cells were seeded at a concentration of 4 × 10^5^ cells/mL in a 10 cm dish and transfected with His-Halo-Rex expression plasmid (10 μg) or His-Halo plasmid (10 μg) as negative control using PEI (polyethyleneimine). After 48 h, cells were collected and whole cell lysate was prepared in Tris-buffered saline containing 0.05% NP-40 (Nacalai Tesque, Inc., Kyoto, Japan) with PIC (protease inhibitor cocktail, Nacalai Tesque, Inc., Kyoto, Japan) (TBSN). Ni-resin (Thermo Fisher Scientific, Inc., Waltham, MA, USA) equilibrated with binding buffer (10 mM Imidazole in TBSN) was added to the whole cell lysate and incubated at 4 °C for 1 h. After washing with the wash buffer (20 mM Imidazole in TBSN), the resin was incubated in elution buffer (500 mM Imidazole in TBSN) at 4 °C for 1 h, then the supernatant was used as the first elution. Next, 40 μL of Halo Resin (Promega, Corp., Madison, WI, USA) equilibrated with TBSN was added to the first elution, incubated at room temperature for 1 h, and washed with TBSN. Finally, 50 μL of SDS-PAGE sample buffer (Fujifilm Wako Chemicals, Corp., Osaka, Japan) was added to the Halo resin, and the proteins interacting with His-Halo-Rex were eluted from the resin by boiling for 5 min as the final elution. Samples at each step were subjected to silver staining (Silver Staining MS Kit, Fujifilm Wako Chemicals, Corp., Osaka, Japan) to confirm purification efficiency and yield. Then, the entire final was subjected to SDS-PAGE with 8% acrylamide gels. The gels were cut out and subjected to in-gel trypsin digestion, then the fragmented peptides were analyzed using the nanoLC-MS/MS system. Gels were cut out in regions containing all molecular weights from 10 kDa to 250 kDa in order to obtain a comprehensive list of proteins interacting with Rex. The mass data (MS/MS spectra) were analyzed by Mascot search (MS/MS Ion Search) to identify the proteins. A total of 81 proteins were identified as interacting with Rex, including those that specifically bound to His-Halo-Rex and those that showed significantly higher binding than nonspecific binding to His-Halo. FEA was performed in those 81 proteins by using STRING v11.5 [11].

### 2.3. Abnormal PD-L1 mRNA Splicing by Rex

In the present study, we focused on PD-L1 mRNA as an mRNA that showed remarkable exon skipping in CEM-Rex compared with CEM-Control in the exon microarray analysis. PD-L1 is a central component of the immune checkpoint machinery that inhibits cytotoxic activity of CD8^+^ cytotoxic T cells (CTLs) by binding to PD-1 expressed on the surface of CTLs. It is abnormally overexpressed in many kinds of tumor cells allowing immune evasion. Therefore, we speculate that overexpression of aberrant *PD-L1* mRNA by Rex, which encodes abnormal PD-L1 mutant protein, may influence the efficiency of immune surveillance of HTLV-1-infected T cells by CTLs. Thus, we specifically focus on the abnormal *PD-L1* mRNA splicing variant found in CEM-Rex. 

#### 2.3.1. Identification of vPD-L1 mRNA Sequence in CEM-Rex

From RNA samples of CEM-Rex and CEM-control cells (*n* = 4) used in the microarray analysis, a cDNA pool was prepared using Super Script II (Invitrogen, Thermo Fisher Scientific, Inc., Waltham, MA, USA). Then, semiquantitative RT-PCR for *PD-L1* mRNA (from exon 3~5) was performed with the following primers and Platinum Taq High Fidelity (Invitrogen, Thermo Fisher Scientific, Inc., Waltham, MA, USA). The PCR-amplified products were separated by 2% agarose gel electrophoresis, and bands were excised to sequence. 

PD-L1 (exon3) For: 5′-GGACCTATATGTGGTAGAGTATG-3′

PD-L1 (exon5) Rev: 5′-CACCAAGGCATAATAAGATGGC-3′

#### 2.3.2. Real-Time q-PCR with Primers Specific for vPD-L1 mRNA

Real-time qPCR was performed on the cDNA samples above. For the detection of *WT-PD-L1* mRNA and *vPD-L1* mRNA separately, the following specific forward primers and a common reverse primer were used (Figure 3C). *ACTB* mRNA was used as an endogenous control.

qWT-PD-L1 For: 5′-GACCACCACCACCAATTCCAAG-3′

qvPD-L1 For: 5′-GTCCTGAGTGG/AGATTAGATCC-3′

qPD-L1 Rev: 5′-CACCAAGGCATAATAAGATGGC-3′

ACTB For: 5′-GCCTGACGGCCAGGTCAT-3′

ACTB Rev: 5′-CAGGACTCCATGCCCAGGAA-3′

#### 2.3.3. NanoLuc Reporter Secretion Assay for sPD-L1

The full-length *WT-PD-L1* cDNA and *vPD-L1* cDNA were amplified by PCR in the above cDNA samples and inserted into the pNL-1 vector (Promega, Corp., Madison, WI, USA). These plasmids express full-length WT-PD-L1 and s(soluble)PD-L1 with NanoLuc at the C-terminal. WT-PD-L1-NanoLuc and sPD-L1-NanoLuc plasmids were transfected into HEK293FT cells using the PEI method, and the amount of PD-L1 in the cells and culture medium was measured at 48 h after transfection by using the NanoLuc assay kit (Promega, Corp., Madison, WI, USA). NanoLuc activity was detected with a Centro LB 960 luminometer (Berthold Technologies GmbH & Co KG, Bad Wildbad, Germany).

#### 2.3.4. Amount of Secreted PD-L1 in HTLV-1-Genome-Expressing Cells

HEK293FT cells were seeded in 6 wells of a 12-well plate at 3 × 10^5^ cells/mL/well, and after 24 h, HTLV-1 infectious plasmid (pX1-MT-M) or pME-18S as negative control was introduced by PEI at 1 μg/well. After 48 h, the amount of secreted PD-L1 in the culture medium was measured by ELISA (Quantikine B7-H1/PD-L1 ELISA Kit, R&D Systems, Inc., Minneapolis, MN, USA).

### 2.4. Biological Significance of the Interaction between Rex and NONO

#### 2.4.1. Construction of the GST-Rex Expression Plasmid

Rex-pME-FLAG [7] was used as a template, and the Rex cDNA fragment was amplified by PCR (Platinum Taq High Fidelity, Invitrogen, Thermo Fisher Scientific, Inc., Waltham, MA, USA) and subcloned into pMEG-2 [12] using EcoRI and NotI site. The primers used for PCR are shown below.

P27Rex (EcoRI) For: 5′-GAATTCATGCCCAAGACCCG-3′

P27Rex (NotI) Rev: 5′-GCGGCCGCTCACATGGGGCAGGAG-3′

#### 2.4.2. GST-Rex Pulldown Assay

HEK293FT cells were seeded at a concentration of 5 × 10^5^ cells/mL and 10 mL/10 cm culture dish one day before transfection. At 24 h after seeding, 10μg pMEG-2 or Rex-pMEG-2 plasmid was transfected by PEI. After 48 h, the cells were collected and whole cell lysate was prepared in TBS buffer containing 1% NP-40 (TBSN). GST-Rex was then collected using Glutathione Sepharose 4G (Cytiva, Marlborough, MA, USA), and the coprecipitated NONO was detected by Western blotting. The bands of GST-Rex and NONO were detected in NBT/BCIP solution (Promega, Corp., Madison, WI, USA). The following antibodies were used in Western blotting. 

Primary antibodies: GST (#Cytiva 27-4577-01, SIGMA-Aldrich, Merck KgaA., Darmstadt, Germany), NONO (#05-950, SIGMA-Aldrich, Merck KgaA., Darmstadt, Germany). 

Secondary antibody: AP (alkaline phosphatase)-conjugated anti-mouse IgG (#S3721), AP-conjugated anti-goat IgG (#V-1151) (both from Promega, Corp., Madison, WI, USA).

#### 2.4.3. NONO Knockdown

In the present study, shRNAs targeting *NONO* mRNA were prepared. Two sets of sense and antisense DNA oligos targeting 3′ UTR and exon 8 of *NONO* and mRNA were annealed by boiling for 5 min, followed by cooling down in room temperature for more than 1 h. Annealed fragments were inserted at BglII/XbaI in pENT4-H1 vector (Invitrogen, Thermo Fisher Scientific, Inc., Waltham, MA, USA). The shNONO fragments in pENT4-H1 were further transferred to the destination vector, CS-RfA-EvBsd (Invitrogen, Thermo Fisher Scientific, Inc., Waltham, MA, USA), by incubation with LR-clonase (Invitrogen, Thermo Fisher Scientific, Inc., Waltham, MA, USA). For preparation of shRNA-expressing lentiviruses, HEK293FT cells were seeded at 1 × 10^5^ cells/mL and 10 mL/10 cm dish. At 24 h after seeding, 10 mg shRNA-CS-RfA-EvBsd, 5 mg CAG-HIV-gp, and 5 mg pCMV-VSV-G/RSV-Rev were co-transfected by PEI method. At 72 h after transfection, the culture medium was filtrated through 45 μm filter and recombinant lentiviruses were precipitated by centrifugation at 10,000 rpm for 3 h at 4 °C. Precipitated viruses were resuspended in 100 μL of RPMI and stored at −80 °C until use. 

For infection, 2 × 10^6^ cells of CEM, MT-2, HUT-102, and TL-Om1 were resuspended in 100 μL shRNA-lentivirus stock, and processed for centrifugation at 2000 rpm for 3 h at 35 °C in order to enhance infection. After centrifugation, cells were resuspended in 2 mL of RPMI (+10% FBS) and incubated at 37 °C with 5% CO_2_ for 72 h before being used for experiments. ShRNA oligos used in the present study are shown below (5′ → 3′).

NONO shRNA-#1

Sense: 5′-ATCCCCGCCAGAGTTCTGCTCTGGAAACGTGTGCTGTCCGTTTCCAGGGTAGAATTCTGGCTTTTTGGAAAT-3′

Antisense: 5′-CTAGATTTCCAAAAAGCCAGAATTCTACCCTGGAAACGGACAGCACACGTTTCCAGAGCAGAACTCTGGCGGG-3′

 

NONO shRNA-#2

Sense: 5′-GATCCCCCAGGTGAAGTCTTCGTTCGTACGTGTGCTGTCCGTATGAATGAAGACTTCGCCTGTTTTTGGAAAT-3′

Antisense: 5′-CTAGATTTCCAAAAACAGGCGAAGTCTTCATTCATACGGACAGCACACGTACGAACGAAGACTTCACCTGGGG-3′

#### 2.4.4. NMD Luciferase Reporter Assay

In this study, we used a highly sensitive and quantitative NMD dual luciferase reporter system by measuring WT- and PTC-containing β-globin mRNA levels [7]. pCDNA6-RSV-Renilla-Luc-β-globin (WT) (Renilla-WT) is expressed independent of NMD in the cell, whereas pCDNA6-RSV-Firefly-Luc-β-globin with PTC in the second exon (Firefly-PTC) has its mRNA targeted by NMD. Therefore, Renilla luciferase activity is not altered by NMD activity, while the firefly luciferase activity would be altered dependent on the cellular NMD activity. NMD activity is therefore calculated as Renilla-luc activity (WT)/firefly-luc activity (PTC). HEK293FT cells were seeded at the concentration of 5 × 10^5^ cells/mL and 10 mL/10 cm culture dish one day before transfection. At 24 h after seeding, 10 μg each of shNONO#1, shNONO#2, and shLuc-CS-RfA-EvBsd was transfected by PEI. After 48 h, the three shRNA-expressing HEK293FT cells were seeded onto 48-well plates at 2 × 10^4^ cells/well, 6 wells each. Then, 24 h later, 100 ng each of Renilla-WT and Firefly-PTC per well was co-transfected by PEI. 24 h later, the activity of Renilla luciferase and firefly luciferase was measured with Dual-Luciferase reporter system (Promega, Corp., Madison, WI, USA) and on Centro LB 960 luminometer (Berthold Technologies GmbH & Co KG, Bad Wildbad, Germany). The knockdown efficiency of shNONO#1 and shNONO#2 was confirmed in HEK293 FT cells by Western blotting. HEK293FT cells, which were transfected with shNONO#1, shNONO#2, or shLuc-CS-RfA-EvBsd were sampled at 48 h after the transfection and lysed in RIPA buffer (10 mM Tris-HCl (pH 7.4), 1% NP-40, 0.1% sodium deoxycholate, 0.1% SDS, 0.15 M NaCl, 1 mM EDTA (pH 8.0)) with protein inhibitor cocktail (Nacalai Tesque, Inc., Kyoto, Japan) and PMSF. Then, the levels of NONO and β-actin were detected by Western blotting with anti-NONO antibody (#05-950, Merck KgaA., Darmstadt, Germany) and β-actin antibody (#sc-69879, Santa Cruz Biotechnology, Inc., Dallas, TX, USA) as primary antibody, and with AP (alkaline phosphatase)-conjugated anti-mouse IgG (#S3721, Promega, Corp., Madison, WI, USA) as the secondary antibody.

#### 2.4.5. RxRE Reporter Assay

Rex specifically binds to the Rex-responsive element (RxRE) in the 3′-UTR of HTLV-1 mRNA and mediates CRM1-dependent nuclear export. To measure the functional activity of Rex, we inserted the RxRE sequence downstream of the β-globin coding region of Renilla-WT (Renilla-WT-RxRE). HEK293FT cells were seeded at 5 × 10^5^ cells/mL and 10 mL/10 cm culture dish one day before transfection. At 24 h after seeding, HEK293FT cells were transfected with 10 μg of shNONO#1, shNONO#2, or shLuc-CS-RfA-EvBsd by PEI. After 48 h, three types of shRNA-expressing HEK293FT cells were seeded onto 48-well plates at 2 × 10^4^ cells/well, 6 wells each, and after 24 h, Renilla-WT-RxRE was transfected at 200 ng per well by PEI. After 24 h, the activity of Renilla luciferase was measured with addition of Renilla substrate (Promega, Corp., Madison, WI, USA) and on Centro LB 960 luminometer (Berthold Technologies GmbH & Co KG, Bad Wildbad, Germany). The protein level of the cell lysate in each well was measured, and the corresponding Renilla activity was normalized by the protein level (mg protein).

#### 2.4.6. Viral Reproduction Assay

HEK293FT cells were seeded in 6 wells of a 12-well plate at 3 × 10^5^ cells/mL/well. After 24 h, HTLV-1 infectious plasmid (pX1-MT-M) or pME-18S as negative control was introduced to 3 wells each at 1 μg/well by PEI. After 48 h, the cells were co-cultured with 1 × 10^5^ cells/well of Jurkat cells containing HTLV-1-LTR driven firefly luciferase gene. After 24 h, LTR activation in Jurkat cells was detected by firefly luciferase assay (Promega, Corp., Madison, WI, USA) on Centro LB 960 luminometer (Berthold Technologies GmbH & Co KG, Bad Wildbad, Germany) as the amount of infectious virus particles. The protein level of the cell lysate in each well was measured, and the corresponding firefly activity was normalized by the protein level (mg protein).

### 2.5. Cell Culture

All cell lines, CEM (T-ALL patient-derived T-cell lines), TL-Om1, HUT102 (ATL patient-derived T-cell lines), MT-2 (HTLV-1-immortalized T-cell lines), and HEK293FT (human embryonic kidney-derived cell line containing the SV40 T-antigen) used in the present study were obtained and maintained as previously reported [13]. CEM, TL-Om1, HUT102, and MT-2 were maintained in RPMI (Gibco, Thermo Fisher Scientific, Inc., Waltham, MA, USA) containing 10% FBS (Gibco, Thermo Fisher Scientific, Inc., Waltham, MA, USA) at 37 °C with 5% CO_2_. HEK293FT was cultured in DMEM (Nissui Pharmaceutical Co., Ltd., Tokyo, Japan) containing 10% FBS (Gibco, Thermo Fisher Scientific, Inc., Waltham, MA, USA) at 37 °C with 5% CO_2_.

The origin of each cell line is as follows. TL-Om1 was provided from Tohoku University, Sendai, Japan. We confirmed that the cell line maintained the characteristics of TL-Om1, i.e., containing one copy of provirus in one cell at the site of 1p13 by fluorescent in situ hybridization and HTLV-1 provirus-specific real-time PCR. MT-2 was provided from Gunma University, Maebashi, Japan. We confirmed the characteristics of MT-2 before use; it contained 10 HTLV-1 proviral cories in one cell and Gag-Tax fusion protein was expressed from a defective provirus of those 10 proviruses. CEM and HUT102 were provided by the Japanese Foundation for Cancer Research (JFCR). We purchased HEK293FT from Riken Cell Bank, Tsukuba, Japan. 

### 2.6. Statistical Analysis

Two-tailed paired Student′s *t*-test was employed to test the statistical difference between the experimental groups throughout the current study. Asterisks in graphs indicate a significant difference between the tested groups (*, *p* < 0.05; **, *p* < 0.01; ***, *p* < 0.001; *n* = 3–6).

## 3. Results

### 3.1. The Effect of Rex on the Gene Expression Profile of T Cells

**Figure 1:**
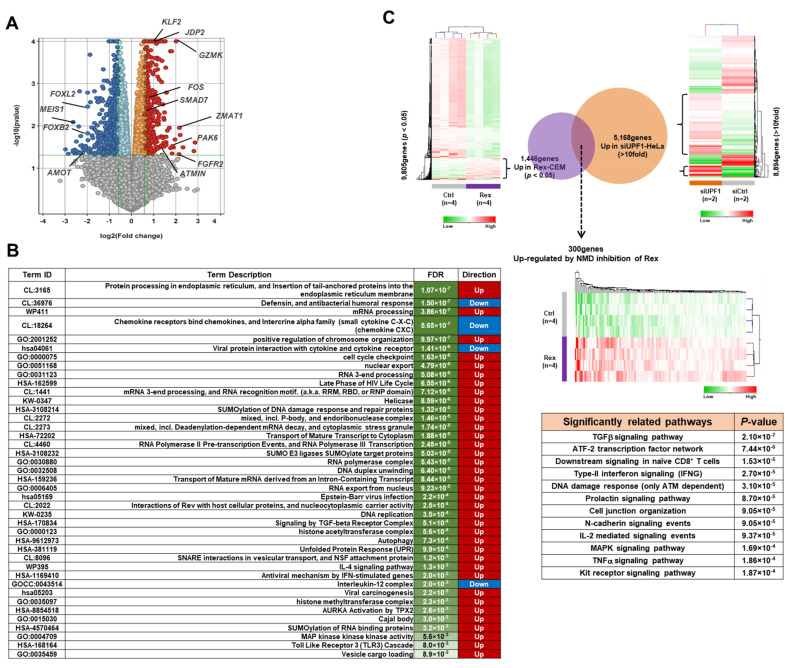
The effect of Rex on the gene expression profile in T cells. (**A**) Results of gene expression microarray analysis in CEM-Rex. Gene expression variation in CEM-Rex relative to CEM-Control is shown by volcano plot. Orange (*p* < 0.05; fold change (Log2) = 0–1), red (*p* < 0.05; fold change (Log2) > 1), light blue (*p* < 0.05; fold change (Log2) = −1–0), blue (*p* < 0.05; fold change (Log2) < −1), grey (not significantly changed). The genes whose expression was significantly and markedly upregulated by Rex included *FOS*, *JDP2*, *GZMK*, *KLF2*, *SMAD7*, *ZMAT1*, *PAX6*, *FGFR2*, and *ATMIN*. On the other hand, the genes with significantly and markedly decreased expression included *MEIS1*, *FOXL2*, *FOXB2*, and *AMOT* (*n* = 4). (**B**) Functional Enrichment Analysis (FEA) of all data on gene expression levels in CEM-Rex against CEM-Control was performed using STRING v11.5. The table lists the pathways significantly associated with genes whose expression was altered in CEM-Rex, where “FDR” is false discovery rate and “Direction” means that most of genes associated with that pathway are significantly upregulated in CEM-Rex. (**C**) Since Rex represses NMD in host cells [7], we expected that some of the mRNAs in CEM-Rex would have increased levels due to the repression of NMD by Rex. In this context, we identified 300 genes that overlap between 1446 genes upregulated in CEM-Rex and 5168 NMD target genes, which were identified by Mendell et al. [14] and are available in Gene Expression Omnibus (GEO, accession number GSE1703), as genes that were upregulated by NMD suppression by Rex. We performed FEA on these 300 genes and listed them in order of *p*-values.

To investigate if Rex alters the gene expression profile of T cells, we conducted gene expression microarray analysis in CEM-Rex and CEM-Control. The result shows that in CEM-Rex, the expression of 1384 genes were significantly (*p* < 0.05) changed by more than 1.5-fold compared to CEM-Control. The upregulated genes included *GZMK*, *FGFR2*, *JDP2*, *KLF2*, *FOS, SMAD7*, *ZMAT1,* and *PAK6*, while the downregulated genes included *MEIS1*, *FOXL2*, *FOXB2,* and *AMOT* (Figure 1A). The changes in gene expression profiles in CEM-Rex compared to CEM-Control were analyzed by Functional Enrichment Analysis to determine their biological effects in cells. The results showed that upregulated genes in CEM-Rex were involved in gene expression regulation and cell proliferation, such as protein processing, mRNA processing, chromatin remodeling, and cell cycle regulation. The downregulated genes, on the other hand, were involved in humoral immune response and chemokine receptor response (Figure 1B). Furthermore, we constructed a Venn diagram using the NMD target mRNA (GEO accession number: GSE1703) in HeLa cells reported by Mendell et al. [14] and a group of genes whose expression was significantly (*p* < 0.05) upregulated in CEM-Rex. There were 300 overlapping genes (Figure 1C). The expression of these genes may have been upregulated by NMD repression by Rex. Pathway analysis of these genes revealed that a significant number of genes are involved in cellular pathways such as TGFβ signaling and ATF2 transcription factor signaling (Figure 1C).

### 3.2. Effect of Rex on the mRNA Splicing Patterns in T cells

**Figure 2:**
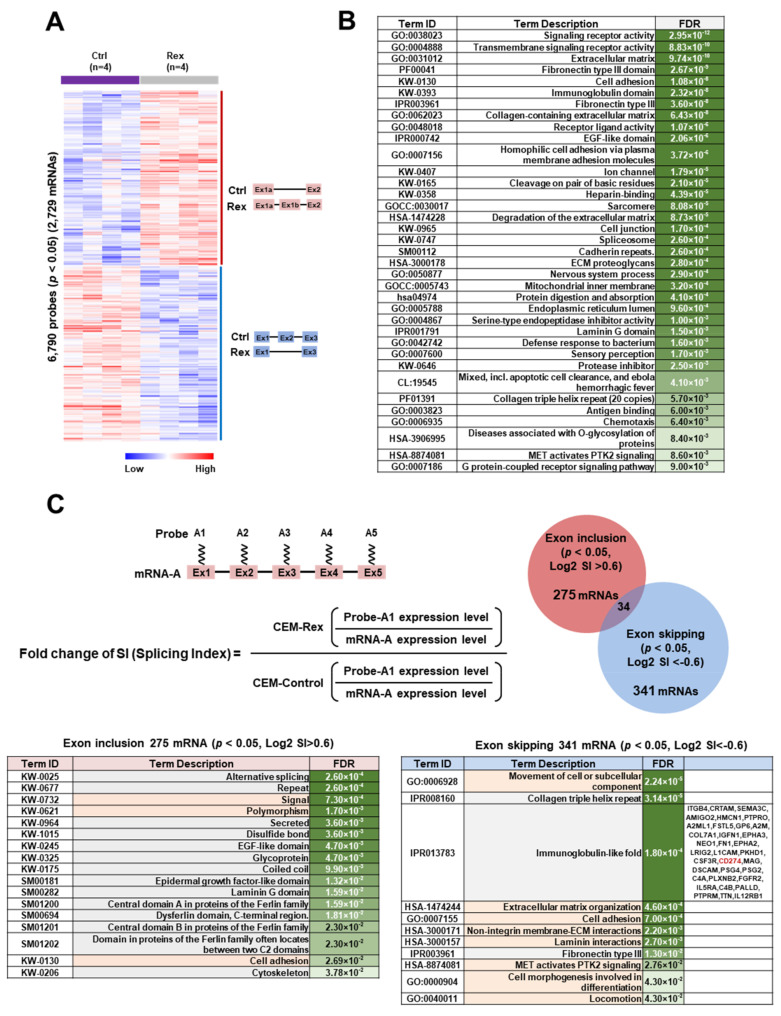
Effect of Rex on the mRNA splicing patterns in T cells. (**A**) Heatmap showing the results of exon microarray analysis in CEM-Rex. The expression levels of 6790 probes in 2729 mRNAs were significantly (*p* < 0.05) changed in CEM-Rex compared to CEM-Control (*n* = 4). (**B**) FEA was performed using STRING v11.5 on 2729 mRNAs that alternatively spliced significantly (*p* < 0.05) in CEM-Rex, and significantly related pathways are listed in order of *p*-values. “FDR” is false discovery rate. (**C**) Splicing index (SI) of exon 1 for a given mRNA-A is expressed as the ratio between the level of exon 1 relative to total mRNA-A expression in CEM-Rex and CEM-Control. One or more SI(s) in 275 mRNAs were significantly increased in CEM-Rex compared with CEM-Control (*p* < 0.05; SI(Log2) > 0.6), i.e., exon inclusion occurred in these mRNAs of CEM-Rex. One or more SI(s) in 341 mRNAs were significantly decreased in CEM-Rex compared with CEM-Control (*p* < 0.05; SI(Log2) < −0.6), i.e., exon skipping occurred in these mRNAs of CEM-Rex. Only 34 mRNAs showed both exon inclusion and exon skipping within a single mRNA. FEA was performed in mRNAs with exon inclusion or exon skipping in CEM-Rex by STRING v11.5. Term IDs related to cellular pathways are indicated by pale orange, and those related to protein structure by pale grey. “FDR” is false discovery rate. Particularly, mRNAs encoding proteins with an immunoglobulin-like fold were significantly abundant in exon skipping. Among them, *CD274* (*PD-L1*) mRNA is shown in red.

Next, we investigated how Rex affects the splicing patterns of mRNAs in T cells by exon microarray analysis in CEM-Rex and CEM-Control. As results, we found that 6790 probes had significantly (*p* < 0.05) altered expression levels compared to controls, and 2729 mRNAs had altered splicing patterns (Figure 2A). Functional Enrichment Analysis (FEA) of these 2729 mRNAs (Figure 2B) revealed that those mRNAs encoded proteins involved in signal transduction, cell adhesion, spliceosome, proteolysis, immune response, and cell migration. Thus, alteration of splicing patterns of those mRNAs by Rex may affect the function and activity of these pathways. Next, we classified the pattern of alternative exon usage according to whether exon skipping or inclusion was occurring. The splicing index (SI) of an exon is defined as the expression level of the exon relative to the total mRNA expression level. If the SI of an exon is high in CEM-Rex relative to CEM-Control, exon inclusion is likely to occur, and if it is low, exon skipping is likely to occur. As shown in the Venn diagram in Figure 2C, there were 275 mRNAs with significant (*p* < 0.05) exon inclusion (Log2 SI > 0.6) and 341 mRNAs with exon skipping (Log2 SI < −0.6). Functional Enrichment Analysis (FEA) was performed on 275 mRNAs in which exon inclusion occurred in CEM-Rex. The results showed that many of these mRNAs encode proteins with EGF-like domains, which function in signal transduction, cell polymorphism, and cell adhesion. Similarly, we performed FEA on 341 mRNAs that undergo Rex-induced exon skipping and found that many of them encode proteins with collagen triple helix repeat and immunoglobulin-like domain. FEA of 341 mRNAs showed that they encode proteins involved in cell migration, adhesion, and extracellular matrix (Figure 2C).

### 3.3. Abnormal CD274 (PD-L1) mRNA Splicing by Rex 

**Figure 3:**
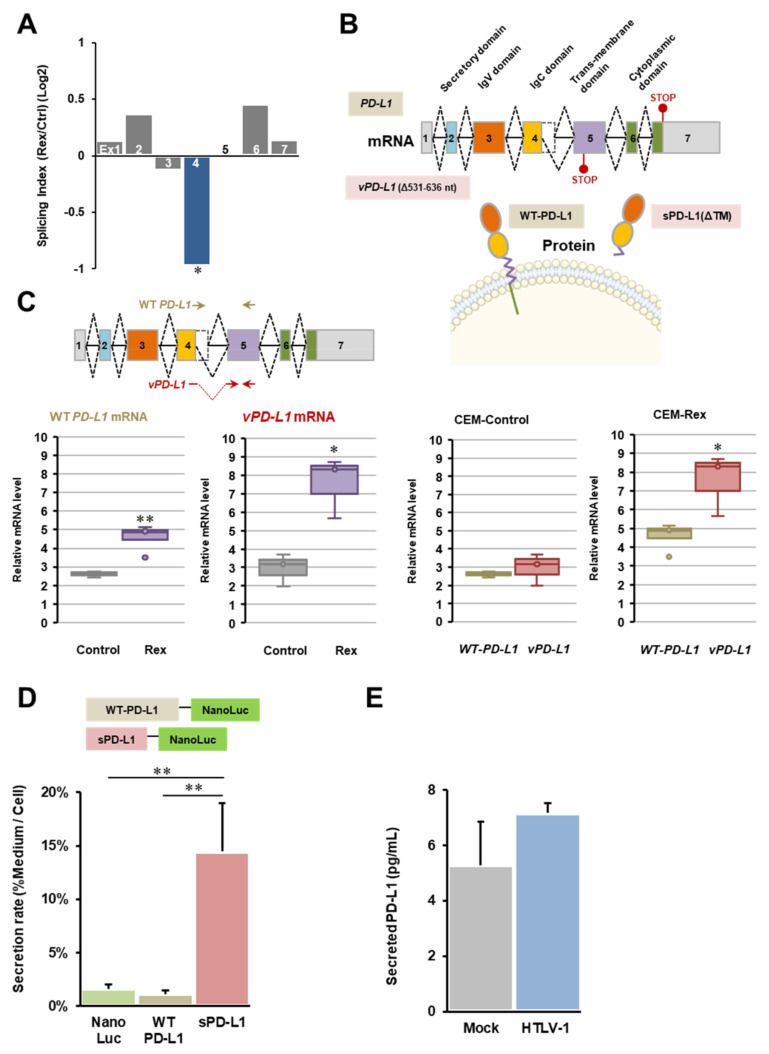
Abnormal *CD274* (*PD-L1*) mRNA splicing by Rex. (**A**) The SI of each exon of *PD-L1* mRNA in the exon microarray analysis of CEM-Rex and CEM-Control is shown in the graph. The SI of exon 4 was significantly decreased in CEM-Rex compared to CEM-Control (*n* = 4; * *p* < 0.05). (**B**) Sequencing analysis of *vPD-L1* mRNA overexpressed in CEM-Rex revealed that 531–636 nt, a part of exon 4, was deleted (*vPD-L1* (Δ531–636)). *vPD-L1* mRNA lacks the cytoplasmic domain downstream of the transmembrane domain due to a premature termination codon (PTC) in exon 5 caused by frameshifting. (**C**) We performed quantitative real-time PCR using primers specific for *WT-PD-L1* mRNA and *vPD-L1* mRNA. Both *WT-PD-L1* mRNA and *vPD-L1* mRNA were significantly upregulated in CEM-Rex compared to CEM-Control, but the level of *vPD-L1* mRNA was more significantly increased in CEM-Rex than in CEM-Control (left two graphs). When *WT-PD-L1* mRNA and *vPD-L1* mRNA expression levels were compared within CEM-Control and CEM-Rex, the amount of *vPD-L1* mRNA was significantly increased only in CEM-Rex (right two graphs) (*n* = 4; * *p* < 0.05; ** *p* < 0.01). (**D**) We overexpressed WT-PD-L1-NanoLuc or sPD-L1-NanoLuc in HEK293FT cells and calculated the secretion rate (% NanoLuc activity in medium/NanoLuc activity in whole cells). The results were compared with HEK293FT expressing only NanoLuc as a negative control. The results showed that the secretion rate of sPD-L1-NanoLuc was significantly and 14-fold higher than that of WT-PD-L1-NanoLuc. In contrast, the secretion rate of WT-PD-L1-NanoLuc was the same as that of NanoLuc. (*n* = 6; ** *p* < 0.01). (**E**) HEK293FT cells expressing the HTLV-1 infectious clone (pX1-MT-M) were cultured to mimic HTLV-1-infected cells, and the amount of sPD-L1 in the supernatant was determined by ELISA. The amount of sPD-L1 in the medium of cells expressing the HTLV-1 plasmid tended to be higher than that in Mock cells, although there was no significant difference (*n* = 3).

Among 341 mRNAs that are alternatively spliced to skip exon(s) in CEM-Rex compared with CEM-Control (*p* < 0.05, Log2 SI < −0.6), we focused on *CD274* (*PD-L1*) mRNA. The splicing index (SI) of the fourth exon of *PD-L1* mRNA in CEM-Rex was significantly decreased (*p* < 0.05), indicating that skipping the exon 4 may occur in *PD-L1* mRNA in CEM-Rex (Figure 3A). In order to confirm the exon microarray results about *PD-L1* mRNA, we sequenced the structure of the *PD-L1* splicing variant (*vPD-L1* mRNA), which is frequently found in CEM-Rex. As a result, it was clarified that a part of exon 4 (531–636 nt) was deleted in *vPD-L1* mRNA. The levels of *WT-PD-L1* mRNA and *vPD-L1* mRNA were then measured by quantitative real-time PCR using primers designed either within the exon4 deletion region or at the newly created junction, respectively. The results showed that the expression of *vPD-L1* mRNA was specifically and significantly upregulated in CEM-Rex (Figure 3C). Finally, we predicted the structure and function of the mutant PD-L1 protein encoded by *vPD-L1* mRNA. The deletion of 531–636 nt causes a frameshift and the appearance of a PTC in exon 5. As shown in Figure 3B, *PD-L1* mRNA consists of seven exons, encoding a secretory domain, an IgV domain encoding a PD-1 binding site, an IgC domain, a transmembrane domain, and an intracellular domain. The mutant PD-L1 encoded by *vPD-L1* mRNA is predicted to form a secreted form (sPD-L1) due to PTC of exon 5 encoding the transmembrane domain causing a loss of the downstream C-terminal region. Therefore, we performed a secretion assay of WT-PD-L1 and sPD-L1 using the NanoLuc assay system. The results showed a 14-fold increase in the secretion rate of sPD-L1 into the extracellular space compared to WT-PD-L1, as expected from its structure. Furthermore, we examined how the expression of sPD-L1 was altered by the expression of the HTLV-1 viral genome by ELISA and found that the amount of sPD-L1 in the culture medium tended to increase in HEK293FT cells transfected with the HTLV-1 infectious plasmid (Figure 3E).

### 3.4. Human Proteins Interacting with Rex in HEK293FT Cells

**Figure 4:**
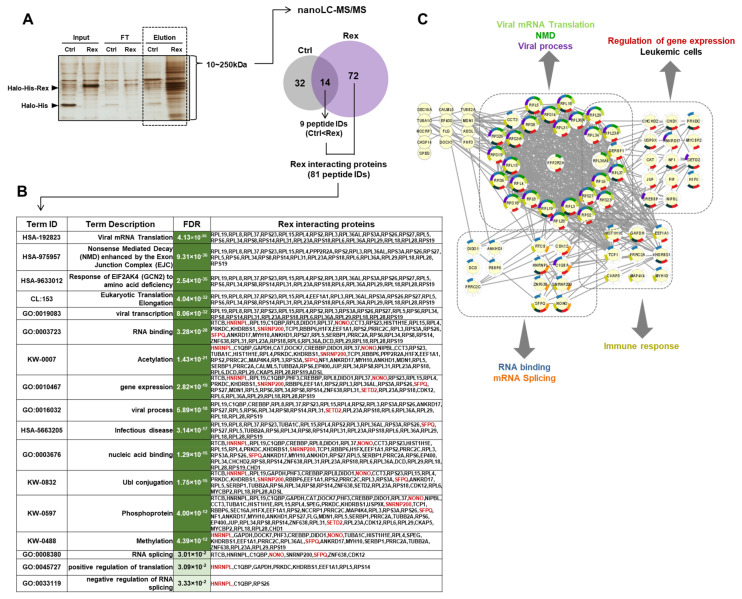
Human proteins interacting with Rex in HEK293FT cells. (**A**) Halo-His-Rex or Halo-His only was overexpressed in HEK293FT cells. Its whole cell lysate (Input) was tandem affinity purified with Ni resin and Halo-tag resin, and the whole size region of the final elution (Elution) was subjected to nano LC–MS/MS. Samples from each step were separated by SDS-PAGE and silver-stained to confirm the enrichment of Halo-His-Rex (left-hand gel photo); FT represents the flow through when the sample was applied to Halo-tag resin. Of the proteins identified by nano LC–MS/MS, 81 were identified as Rex-interacting proteins: 72 detected only in Halo-His-Rex and 9 detected also in Halo-His but in significantly higher amounts in Halo-His-Rex (right-hand Venn diagram). (**B**) The 81 proteins interacting with Rex were subjected to FEA using STRING v11.5 and significantly related pathways are listed in order of *p*-values. “FDR” is false discovery rate. Proteins involved in regulation of gene expression and RNA splicing, including NONO, SFPQ, SETD2, hnRNPL, and snRNAP200, are highlighted in red. (**C**) Pathway analysis in 81 proteins was performed using STRING v11.5 and visualized by Cytoscape (an open-source software developed by Cytoscape Consortium). Many of the proteins were divided into groups related to viral mRNA translation/NMD/viral process, regulation of gene expression/leukemic cells, RNA binding/mRNA splicing, and immune response.

To identify the human cellular proteins interacting with Rex, we performed tandem affinity purification of ectopically expressed His-Halo-tagged Rex in HEK293FT cells and identified co-precipitated cellular proteins by the nano LC–MS/MS system. As a result, we identified 81 human intracellular proteins that interact with Rex (Figure 4A). We performed FEA on these 81 proteins and found that they are involved in viral mRNA translation, NMD, viral transcription, RNA binding, viral process, infectious disease, protein post-translational modification, and RNA splicing (Figure 4B,C). In particular, Rex was found to interact with a number of ribosomal and RNA-binding proteins to regulate the nuclear export and translation of viral mRNAs. In addition, Rex interacted with proteins involved in gene expression regulation and mRNA splicing, confirming that Rex has a function in influencing gene expression and splicing patterns, as shown in Figure 1 and Figure 2, respectively.

### 3.5. Interaction between Rex and NONO

**Figure 5:**
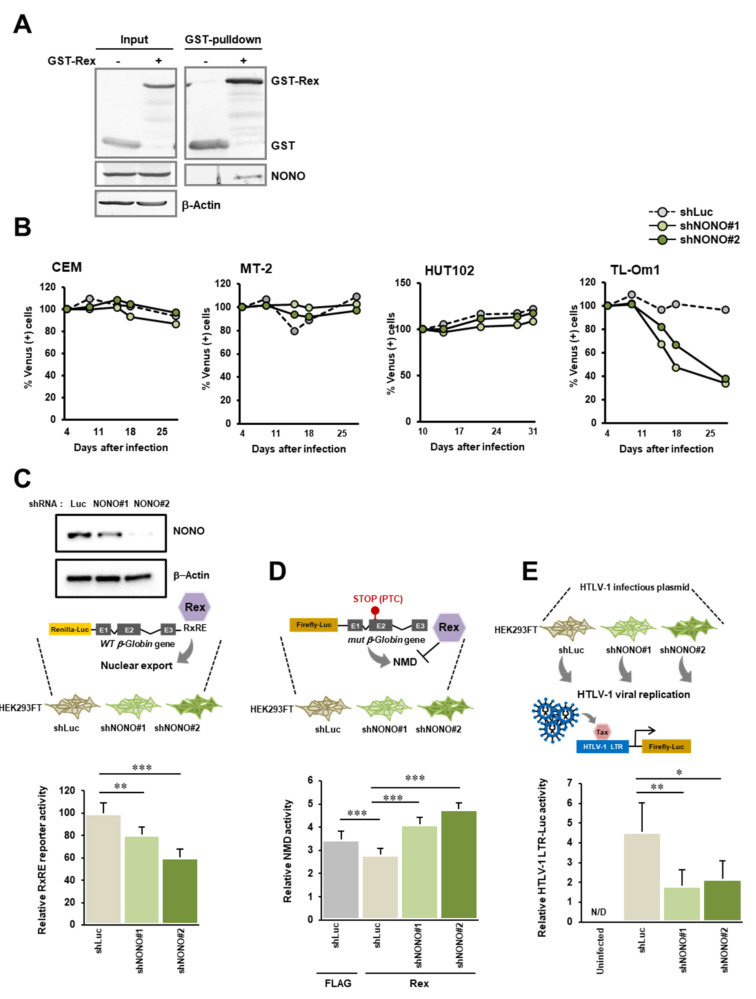
Interaction between Rex and NONO. (**A**) Whole cell lysate of HEK293FT cells overexpressing GST-Rex was purified with glutathione Sepharose, and NONO coimmunoprecipitated with GST-Rex was detected by Western blotting. In the case of GST alone, no NONO band was detected, confirming the specific interaction between Rex and NONO. (**B**) The effect of NONO knockdown on cell viability in CEM, MT-2, HUT102, and TL-Om1 was investigated. Since cells expressing shNONO#1 or shNONO#2 by the lentiviral expression system express Venus, the percentage of Venus (+) cells, i.e., NONO knockdown cells, was measured chronologically by flow cytometer and the viability of NONO knockdown cells (%) was calculated. (**C**) NONO knockdown HEK293FT cells were transfected with Rex and WT-Glo-RxRE reporter plasmid. NONO knockdown significantly reduced Renilla firefly activity, which indicates RxRE-dependent nuclear export efficiency was significantly reduced in NONO knockdown HEK293FT cells. (*n* = 6, mean ± SD, ** *p* < 0.01, *** *p* < 0.001). The upper panel shows the levels of NONO in HEK293FT cells expressing shNONO#1 and shNONO#2. (**D**) NONO knockdown HEK293FT cells were transfected with NMD activity reporter plasmids, Renilla-WT, and Firefly-PTC, and the NMD was calculated as (Renilla luciferase activity/firefly luciferase activity). The significant suppression of NMD by Rex, which was cancelled by NONO knockdown, suggests that NONO plays an essential role in the suppression of NMD by Rex. (*n* = 6, mean ± SD, *** *p* < 0.001). (**E**) NONO knockdown HEK293FT cells were transfected with an HTLV-1-infecting plasmid (pX1-MT-M) to investigate the relationship between NONO and the efficiency of HTLV-1 virus particle production. The efficiency of HTLV-1 virus particle production was determined by co-culturing with Jurkat cells containing the firefly luciferase gene regulated by HTLV-1 LTR for 48 h. The NONO knockdown significantly reduced the firefly luciferase activity, indicating that NONO promotes the production of viral particles from the HTLV-1 genome. (*n* = 3, mean ± SD, * *p* < 0.05, ** *p* < 0.01).

Among 81 proteins that have been shown to interact with Rex, we focused on NONO, a multifunctional protein involved in gene expression regulation and mRNA splicing in the nucleus, along with SFPQ [15], which has also been shown to interact with Rex. We first reconfirmed the interaction between NONO and Rex in cells by GST-Rex pulldown assay (Figure 5A). We also confirmed the subcellular co-localization and the interaction between Rex and NONO in the nucleus of HeLa cells (Figure S1). Then, we overexpressed shRNA against NONO by lentiviral expression system and examined the relationship between NONO and cell proliferation ability in various cell lines. We found that NONO knockdown had no effect on cell viability in CEM (HTLV-1−), MT-2 (HTLV-1 + /Rex +), and HUT102 (HTLV-1 + /Rex+). In contrast, in TL-Om1 (HTLV-1 +/Rex−), a cell line derived from tumor cells of an ATL patient, there was a significant decrease in cell viability of NONO knockdown cells (Figure 5B). The cellular expression level of NONO is relatively high in CEM, MT-2, and HUT102, while it is somewhat lower in TL-Om1 (Figure S2). We further examined the effect of NONO on Rex function. We first examined the relationship between RxRE-dependent mRNA nuclear export by Rex and NONO. NONO knockdown significantly reduced RxRE reporter activity, i.e., the efficiency of RxRE-dependent mRNA nuclear export by Rex is supported by NONO (Figure 5C). We next examined the effect of NONO on Rex-mediated NMD repression and found that NONO knockdown cancelled Rex-mediated NMD repression (Figure 5D). Finally, we examined the effect of NONO on the production of viral particles from HTLV-1 infectious plasmids. We found that NONO knockdown significantly reduced the efficiency of infectious HTLV-1 particle production (Figure 5E). These results suggest that NONO may promote Rex function and HTLV-1 viral replication.

## 4. Discussion

In the present study, we investigated the transcriptome and interactome of HTLV-1 Rex, as well as its effects on the splicing pattern of human T cells for the first time. Integrated analysis of those data demonstrated that Rex interacts with cellular proteins involved in the host gene expression processes and Rex changes the gene expression profile and mRNA splicing patterns in T cells, probably by interacting with those cellular proteins.

### 4.1. Impact of Rex on Gene Expression Profiles Icluding AP-1 Family Proteins

Our results show that the gene expression profile of Rex-expressing T cells is dynamically altered. In particular, the expression of genes encoding AP-1 family proteins, such as *FOS* and *JDP2*, was significantly upregulated (Figure 1A). AP-1 is a transcription factor formed by homo- or heterodimers of the AP-1 family proteins belonging to the subfamily of JUN (v-JUN, c-JUN, JUNB, and JUND), FOS (v-FOS, c-FOS, FOSB, and FRA1,2), MAF (c-MAF, MAFB, MAFA, MAFG, MAFF, and MAFK), and ATF (ATF2–7, ATF3/LRF1, B-ATF1–3, JDP1, and JDP2). AP-1 plays an essential role in the function and differentiation of immune cells, through the regulation of cell proliferation and apoptosis [16,17]. Therefore, dysregulation of the AP-1 pathway is thought to have a major impact on the oncogenic and cancer immune status of cells [18,19]. Recently, AP-1 has been found to play an important role in chromatin opening and gene expression regulation during T cell activation [20]. Moreover, it was reported that BAF chromatin-remodeling complex binds to AP-1 on the genome to enhance opening the enhancer region and activating gene expression [21]. Many oncoviruses are also thought to interfere with the AP-1 pathway in host cells, promoting immune response evasion in infected cells and triggering oncogenesis [22]. In HTLV-1-infected cells, Tax activates the AP-1 pathway by upregulating the expression of key AP-1 family proteins such as c-FOS, FRA-1, c-JUN, JUNB, and JUND. HBZ binds repressively to c-JUN and JUN and reduces AP-1 activity, while increasing its transcriptional activity through interaction with JUND [23,24]. As described above, HTLV-1 Tax and HBZ have been thought to regulate AP-1 activity in infected cells and contribute to their survival, proliferation, and oncogenesis [25]. In the present study, we showed that Rex, like Tax, upregulates *FOS* expression (Figure 1A). Rex also upregulated the expression of *JDP2*, which functions as a repressor of JUN (Figure 1A), indicating that Rex may be involved in both positive and negative regulation of AP-1 activity. How Rex functions in concert with Tax and HBZ to regulate the AP-1 pathway will be investigated in the future, especially in a setting with these viral proteins together.

Functional Enrichment Analysis (FEA) of gene expression profiles in CEM-Rex cells showed that upregulated genes were involved in chromatin organization, mRNA processing, and protein translation and modification. On the other hand, downregulated genes were involved in humoral immunity, chemokine receptors, and IL-12 pathway (Figure 1B). It was found that Rex positively regulates not only the pathways involved in its function as an RNA-binding protein, but also gene expression regulation, affecting a variety of pathways in the host cell. In addition, the expression of host factors with which the HIV-1 counterpart Rev interacts, as well as of genes that vary with viral infection, such as EBV and HIV-1, was significantly altered in CEM-Rex. Furthermore, antiviral mechanisms stimulated by IFN-stimulated genes and the TLR3 cascade were positively regulated, indicating that Rex triggers part of the viral infection response in host cells. In CEM-Rex, AURKA and MAPK pathways, as well as vesicular transport were activated, suggesting that Rex not only promotes viral particle replication but is also involved in its assembly and release. Finally, genes involved in viral carcinogenesis were also upregulated, suggesting that the action of Rex may trigger tumorigenesis in host cells.

We previously reported that Rex suppresses the host mRNA quality control mechanism NMD (nonsense-mediated mRNA decay) and stabilizes HTLV-1 genomic RNA [7]. We therefore extracted 300 genes whose expression was significantly upregulated by Rex among NMD target mRNAs, which were identified by Mendell et al. [14] and are available in Gene Expression Omnibus (GEO, accession number GSE1703), and performed Functional Enrichment Analysis (FEA) of these 300 genes (Figure 1C). The results showed that genes involved in TGF-β, ATF2, IFNγ, N-cadherin, IL-2, MAPK, TNFα, and Kit receptor pathways were significantly accumulated. These results suggest that Rex may affect the activity of these pathways by repressing host NMD.

### 4.2. Effect of Rex on mRNA Splicing Patterns in T Cells

The mRNA splicing is a complex mechanism involving a large complex formed by spliceosomes and more than 100 splicing factors [26]. The functions and activities of these spliceosomes and splicing factors are fine-tuned in a tissue-specific manner. The expression of multiple alternative splice variants from the same gene results in variety in the function and activity of the encoded protein, enabling tissue-specific cell differentiation [27,28]. Therefore, dysfunctions in the splicing mechanism can lead to abnormal cell phenotypes and tumorigenesis [29,30,31,32].

Rex specifically binds to RxRE in the 3′UTRs of unspliced (*Gag*/*Pol*) mRNA and incompletely spliced (*Env*) mRNA of HTLV-1 and transports these unstable viral mRNAs out of the nucleus in a CRM1-dependent manner, allowing translation of viral structural proteins and replication of viral particles [4,5]. However, the mechanism by which Rex avoids splicing occurring simultaneously with transcription is not known. Recently, chromatin signatures have also been identified that specify the rate of exon inclusion or skipping [33], clarifying the link between epigenetic regulation and splicing regulation. We hypothesized that Rex intervenes in this highly organized host cell splicing machinery and influences its activity to nuclear export HTLV-1 unspliced and partially spliced mRNAs. Thus, we analyzed the changes in splicing patterns in Rex-expressing T cells. We found that alternative exon usage occurred in 2729 mRNAs (Figure 2A), indicating for the first time that Rex has a major impact on the splicing pattern of a wide range of mRNAs in T cells. Functional Enrichment Analysis (FEA) of 2729 mRNAs whose splicing patterns were altered by Rex showed that mRNAs encoding proteins involved in cell membrane receptors, cell adhesion, and cell migration were significantly enriched. This suggests that the altered splicing pattern by Rex may affect these pathways by translating isoforms that are not normally expressed. In addition, Rex tended to include the exon of mRNAs encoding proteins with an EGF-like domain and, conversely, tended to skip the exon of mRNAs encoding proteins with an Ig-like domain (Figure 2B). Since the repeat sequences of each domain may be encoded by similar mRNA motifs, Rex may be interacting with factors involved in splicing near these mRNA motifs. Details remain to be studied.

### 4.3. Physiological Effect of Abnormal Splicing of PD-L1 mRNA by Rex

In the present study, we specifically investigated the biological impact of abnormal *PD-L1* mRNA in CEM-Rex (Figure 3). PD-L1 plays a central role to establish immune-tolerance against self, which is called the immune checkpoint machinery. PD-L1 is expressed by antigen-presenting cells (APCs) such as dendritic cells and macrophages. PD-L1 on those APCs binds to PD-1 expressed on CD8^+^ cytotoxic T cells (CTLs) to inhibit its cytotoxic activity. It is well known that PD-L1 is also abnormally overexpressed in many kinds of tumor cells allowing immune evasion. Alterations in the expression of PD-L1 and PD-1 have also been reported in virus-infected cells, suggesting that it promotes immune evasion in infected cells by manipulating CTL activity [34,35]. In this study, we found overexpression of *vPD-L1* mRNA (Δ531–636 nt) with partial loss of exon 4 in CEM-Rex cells (Figure 3A,C). This variant has been registered with NCBI as *PD-L1* mRNA variant 3 (NR_052005). A premature termination codon (PTC) was generated in *vPD-L1* mRNA at exon 5 and encodes a secreted form of sPD-L1 that lacks the transmembrane domain and the cytoplasmic domain (Figure 3B). Indeed, a 14-fold increase in the rate of extracellular secretion was observed with sPD-L1 compared with WT-PD-L1 (Figure 3D). In the culture supernatant of HEK293FT cells expressing the HTLV-1 genome, the amount of secreted PD-L1 tended to increase (Figure 3E). Since sPD-L1 maintains its IgV/IgC domains and can bind to PD-1, the release of sPD-L1 from infected cells by Rex may inhibit CTL activity without cell-to-cell contact, facilitating immune escape of the infected cells.

### 4.4. Rex May Affect the Gene Expression Profile and mRNA Splicing Patterns via Interation with Various Cellular Proteins 

In this study, we identified 81 Rex-interacting proteins (Figure 4A). We performed FEA on these proteins and found that they include proteins involved in NMD and RNA binding, confirming that the function of Rex as a viral RNA-binding protein [4,5] and NMD repressor [7] is mediated by its interaction with these host cellular proteins. The interaction of Rex with a number of ribosomal proteins indicates that Rex intervenes in the protein translation pathway. Furthermore, interactions between Rex and proteins involved in gene expression and mRNA splicing were observed. These results strongly suggest that Rex has a novel function in regulating these mechanisms. The changes in gene expression profiles and splicing patterns shown in Figure 1 and Figure 2 of this study may be the result of these novel Rex functions (Figure 4B). In particular, Rex interacts with NONO, SFPQ, shRNP200 (U5), a spliceosome, hnRNPL, and SETD2, which specifically methylate K36 of histone H3 (H3K36me3) [36]. H3K36me3 is known to be a chromatin marker that regulates alternative exon usage [37]. Recently, it was reported that SETD2 regulates the deposition of H3K36me3 by interacting with hnRNPL [38], and that SETD2 suppresses metastatic progression of prostate cancer by methylating EZH2 to lead its degradation [39]. Furthermore, it has been reported that H3K36me3 epigenetic status by SETD2 is essential for the maintenance of productive viral replication and late viral mRNA splicing in the HPV lifecycle [40]. In HTLV-1-infected cells, Rex may intervene in the interaction between SETD2 and hnRNPL to modify the positioning of H3K36me3, thereby regulating gene expression and alternative exon usage of the viral gene to maintain unspliced/partially spliced viral mRNAs. In addition, Rex selectively interacts with host factors associated with viral translation, viral transcription, viral process, and infectious diseases, rather than with general translation and transcription factors (Figure 4B). Rex may selectively interact with host proteins particularly involved in the transcription and translation of the viral genome, to favor the expression of the HTLV-1 genome. Interactions with factors involved in splicing and NMD regulation also provide evidence for a potential mechanism of Rex that prevents unspliced/partially spliced HTLV-1 mRNAs from undergoing unnecessary splicing and degradation (Figure 4C). The limitation of those data is, however, that the interactome analysis was conducted in HEK293FT cells, but not in T cells. It is necessary to also analyze the interaction of Rex and human proteins, especially in regard to notable factors discussed above, in Rex-expressing T cells, such as MT-2 (HTLV-1 immortalized T-cell line) and HUT102 (ATL patient-derived T-cell line). 

### 4.5. The Biological Importance of the Interaction between Rex and NONO

Our results suggest that Rex may intervene and influence various mechanisms in human T cells, including gene expression, splicing, mRNA quality control, protein translation, and protein modification, in order to fulfil its function of nuclear export of HTLV-1 mRNA. We focused on the interaction with NONO (the non-POU domain-containing octamer-binding protein NONO/p54nrb) as a cellular factor that confers various functions on Rex (Figure 4B and Figure 5A). Our results indicate that the interaction between Rex and NONO promotes Rex functions such as RxRE-dependent mRNA nuclear export (Figure 5C) and NMD inhibition (Figure 5D). In addition, NONO was shown to promote viral particle replication from the HTLV-1 genome, indicating that NONO is involved in maintaining Rex function, which is essential for viral genome expression and particle replication from the provirus (Figure 5E). NONO belongs to the Drosophila behavior/human splicing (DBHS) family and was first identified as a splicing factor in mammalian cells [15,41]. Subsequently, it has been shown that NONO is involved not only in splicing but also in gene expression regulation, mRNA processing, gene damage response, cell proliferation, apoptosis, migration, and various other cellular functions [15,41]. Recently, it has been shown that nuclear paraspeckle formation by the histone modifier ASXL1 and the function of NONO in the nuclear paraspeckle are essential for normal differentiation from hematopoietic stem and progenitor cells (HSPCs) [42]. NONO often forms heterodimers with SFPQ (splicing factor proline/glutamine rich), another human DBHS family protein, and exerts its function [15,41]. The NONO/SFPQ heterodimer has been reported to be responsible for maintaining telomere stability in human cells [43]. As NONO is a multi-functional protein involved in various cellular pathways, its abnormal function has been reported to be involved in the proliferation and malignant phenotypes of various tumor cells [41,44,45]. Since NONO and SFPQ are closely related to the regulation of immunocompetent cell functions, it has been reported that the interaction of various virus factors with NONO and/or SFPQ in infected cells affects the efficiency of viral replication [15]. For example, SFPQ/PSF is essential for effective influenza virus transcription by increasing viral mRNA polyadenylation [46]. EBER2, which is encoded by EBV and expressed in latently infected cells, interacts with PAX5 via NONO and SFPQ and regulates the expression of viral latent membrane proteins 1 and 2 (LMP1/2) [47]. It was also reported that oriP, a new EBV noncoding RNA recently identified, promotes viral gene expression in lytic infections and regulates the innate immune response by interacting with NONO to maintain viral replication [48]. In particular, in HIV-1, which is closely related to HTLV-1, NONO interacts with the viral preintegration complex (PIC) and inhibits infection of CD4^+^ T cells [49], while SFPQ is essential for RRE (Rev responsive element)-dependent transport of unspliced viral mRNA by HIV-1 Rev [50]. HTLV-1 Rex and HIV-1 Rev have overlapping functions in CRM1-dependent nuclear export of viral unspliced mRNA [4]. In the present study, the interactions between Rex and NONO/SFPQ have been confirmed (Figure 4B). Since NONO and SFPQ often function as heterodimers, Rex may bind to NONO/SFPQ heterodimers and regulate various functions of these multifunctional DBHS proteins favorable for viral replication, thereby affecting gene expression and splicing patterns.

### 4.6. Summary: New Possibilities in the Rex Functional Pathways in HTLV-1-Infected T Cells

**Figure 6:**
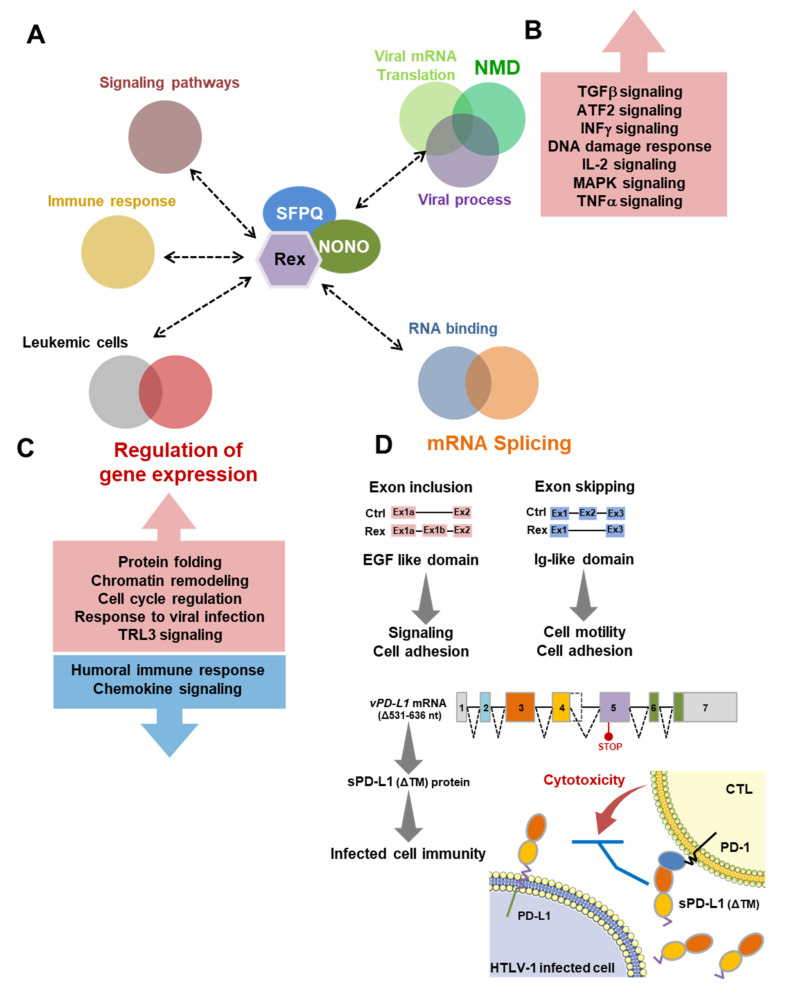
New possibilities in the Rex functional pathways in HTLV-1-infected T cells. (**A**) Our results show that Rex interacts with proteins in human cells involved in gene expression, mRNA translation, NMD, viral process, RNA binding, mRNA splicing, immune response, and various signaling pathways. In particular, through NONO and SFPQ, Rex may influence various pathways in the cell. (**B**) The NMD repression by Rex results in the upregulation of genes involved in TGFβ, ATF2, INFγ, DNA damage response, IL-2, MAPK, and TNFα signaling whose expression levels are originally regulated by NMD. (**C**) Rex has been shown to upregulate genes encoding proteins involved in protein folding, chromatin remodeling, cell cycle regulation, response to viral infection, and TRL3 signaling, and suppress those involved in humoral immune response and chemokine signaling. (**D**) Rex influences the splicing pattern of many mRNAs. Exon inclusion tends to occur in mRNAs encoding EGF-like domain-containing proteins involved in signaling and cell adhesion, while mRNAs that are prone to exon skipping encode Ig-like domain-containing proteins involved in cell motility and cell adhesion. In particular, the amount of *vPD-L1* (Δ531–636nt) mRNA was significantly elevated in CEM-Rex. Since this variant encodes a soluble PD-L1 (sPD-L1) lacking the transmembrane domain, early HTLV-1-infected T cells expressing Rex may release sPD-L1, which binds to PD-1 on CD8^+^ CTLs without cell-to-cell contact in the surrounding microenvironment and manipulates the immune response against infected cells.

In this study, for the first time, we have comprehensively analyzed the transcriptome and alternative splicing profiles in Rex-overexpressing T cells, together with Rex interactome to elucidate novel functions and effects of Rex in infected T cells (Figure 6). Although Rex is known to transport unspliced and partially spliced HTLV-1 mRNAs out of the nucleus in an RxRE-dependent manner, the mechanism by which it does so has not been investigated in detail. Our results suggest that Rex may intervene in the regulation of gene expression and splicing through interaction with multifunctional host proteins such as NONO and SFPQ (Figure 6A). Rex also interacts with a number of ribosomal proteins, suggesting that it is involved in the regulation of the host cell translational system and its co-evolving NMD (Figure 6A). We have previously reported that Rex represses NMD [7]. In the present study, we found 300 NMD target mRNAs were upregulated in CEM-Rex, suggesting that NMD inhibition by Rex may have stabilized those NMD-regulated mRNAs, which encode proteins in various signaling pathways (Figure 6B). We show that Rex intervenes in various steps of the T cell gene expression pathway to create a favorable intracellular environment for viral replication. On the other hand, such a function of Rex may significantly alter T cell gene expression patterns (Figure 6C) and splicing patterns (Figure 6D), affecting various downstream intracellular pathways and phenotypes. In particular, the increased expression of *vPD-L1* mRNA by Rex and the consequent release of sPD-L1 from infected cells may regulate the activity of surrounding CTLs, favoring immune escape of infected cells (Figure 6D).

In summary, Rex was shown for the first time to strictly regulate viral replication by intervening in various pathways in host T cells, escorting HTLV-1 mRNA from transcription to translation. In turn, such function of Rex may alter gene expression and splicing patterns, affecting the phenotype and function of infected cells. Our next study will examine the function of Rex in the presence of other important HTLV-1 accessory proteins, such as Tax and HBZ, to more realistically assess its function and impact in HTLV-1-infected cells.

## Data Availability

The microarray data obtained in the present study are available in the following database. (1) Human exon microarray analysis in CEM cells overexpressing HTLV-1 Rex GEO (GSE193574) https://www.ncbi.nlm.nih.gov/geo/query/acc.cgi?acc=GSE193574; (2) Human gene expression microarray analysis in CEM cells overexpressing HTLV-1 Rex GEO (GSE193576) https://www.ncbi.nlm.nih.gov/geo/query/acc.cgi?acc=GSE193576.

## Supplementary Materials

The following supporting information can be downloaded at: https://www.mdpi.com/article/10.3390/v14020407/s1, Figure S1. Subcellular co-localization and interaction between Rex and NONO, Figure S2. NONO expression levels in various T-cell lines.
