# Supplementary material for: Exploring New Functional Aspects of HTLV-1 RNA-Binding Protein Rex: How Does Rex Control Viral Replication?
Source: Viruses. 2022 Feb 16;14(2):407. doi: 10.3390/v14020407 (PMC8877913; doi:10.3390/v14020407)
Supplement: Supplementary file 1 [file viruses-14-00407-s001.zip › viruses-1556671-supplementary.pdf]

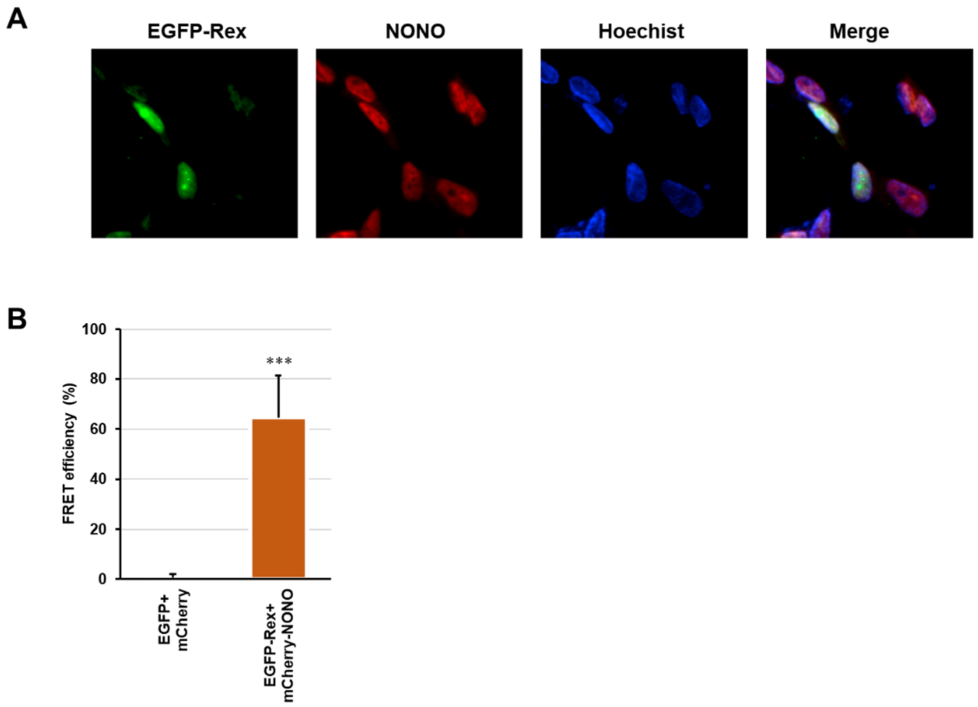

Figure S1. Subcellular co-localization and interaction between Rex and NONO.
A. Subcellular localization of Rex and NONO in HeLa cells. HeLa cells were seeded at $3x10^5$ cells/mL and 200 μL/well of a 4-well culture slide glass. After 24h, 1μg of Rex-pEGFP-C1 was transfected by PEI method. After further 24 h, cells were fixed with 4% paraformaldehyde followed by blocking in the blocking buffer (5% BSA in PBS). Cells were immuno-stained with anti-NONO antibody (#05-950, Merck KgaA., Darmstadt, Germany) and goat anti-mouse IgG conjugated with Alexa Fluor 546 (#A-11035, Invitrogen, Thermo Fisher Scientific, Inc., Waltham, MA, USA). Cells were sealed in the mounting medium (80% glycerol containing Hoechist 33258). The subcellular localizations of Rex and NONO were observed by Olympus iX73 fluorescent microscope (Olympus, Corp., Tokyo, Japan). The results show that Rex and NONO colocalize in nucleus. **B.** Subcellular interaction between Rex and NONO was examined by FRET (fluorescence resonance energy transfer). In FRET, an energy transfer from donor (EGFP-Rex) to acceptor (mCherry-NONO) occurs only when the distance between these molecules is less than 10 nm in which they are highly likely physically interacting. HeLa cells were seeded at $3x10^5$ cells/mL and 200 μL/well of a 4-well culture slide glass. After 24 h, 500ng each of Rex-pEGFP-C1 and NONO-pmCherry-C1 (both vectors were purchased from Clontech, Takara Bio Inc., Shiga, Japan), or pEGFP-C1 and pmCherry-C1 (the negative control), were co-transfected to HeLa cells using Lipofectamine2000 (Invitrogen, Thermo Fisher Scientific, Inc., Waltham, MA, USA). After further 24 h, cells were fixed in 4% paraformaldehyde, and sealed in the mounting medium (80% glycerol containing DAPI). Then the changes of the EGFP intensity before and after the photobleaching of mCherry were detected by LSM710 confocal microscope (Carl Zeiss, AG., Oberkochen, Germany). Photobleaching was conducted in 6 regions of nuclei in each group of HeLa cells expressing EGFP-Rex and mCherry-NONO or EGFP and mCherry (n = 6). The FRET efficiency was calculated as [1-(average of pre-photobleaching intensity of EGFP) / (average of post-photobleaching intensity of EGFP)] ×100 (%). The graph shows the FRET efficiency (%) in HeLa cells expressing EGFP-Rex and mCherry-NONO or EGFP and mCherry (n = 6, Mean ± SD, ***; $p < 0.01$ in two-tailed Student’s *t*-test). The results demonstrated that Rex and NONO interact in nuclei of HeLa cells.

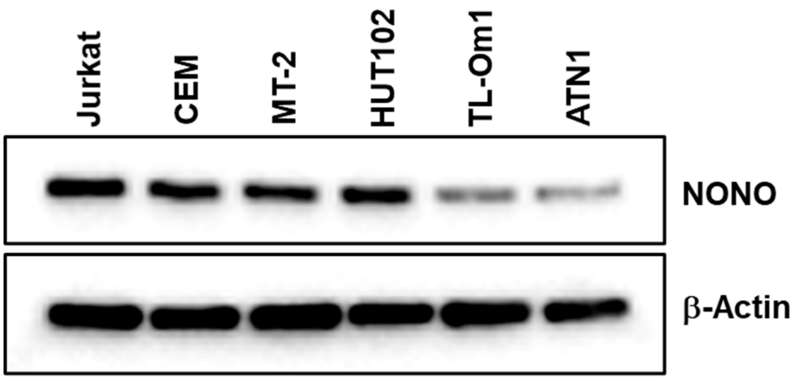

Figure S2. NONO expression levels in various T-cell lines.
NONO expression levels were examined in various T-cell lines such as Jurkat, CEM (TALL patient derived T-cell lines), MT-2 (HTLV-1 immortalized T-cell line), HUT102 (ATL patient derived T-cell line with viral protein expressions), TL-Om1, and ATN1 (ATL patient derived T-cell line without viral protein expressions). The Western blotting of whole cell lysate samples in RIPA buffer from those cell lines showed relatively high expression levels of NONO in Jurkat, CEM, MT-2, and HUT102. On the other hand, the NONO level is lower in TL-Om1 and ATN1. The NONO antibody (#05-950, Merck KgaA., Darmstadt, Germany) and β-Actin antibody (#sc-69879, Santa Cruz Biotechnology, Inc., Dallas, TX, USA) was used as primary antibody and AP (alkaline-phosphatase) conjugated anti-mouse IgG (#S3721, Promega, Corp., Madison, WI, USA) was used as the secondary antibody in the Western blotting.
